# Correlation Between Periostin Expression and Pro-Angiogenic Factors in Non-Small-Cell Lung Carcinoma

**DOI:** 10.3390/cells13171406

**Published:** 2024-08-23

**Authors:** Adrian Wasik, Marzenna Podhorska-Okolow, Piotr Dziegiel, Aleksandra Piotrowska, Michal Jerzy Kulus, Alicja Kmiecik, Katarzyna Ratajczak-Wielgomas

**Affiliations:** 1 Division of Histology and Embryology, Department of Human Morphology and Embryology, Wroclaw Medical University, 50-368 Wroclaw, Poland; adrian.wasik@student.umw.edu.pl (A.W.); piotr.dziegiel@umw.edu.pl (P.D.); aleksandra.piotrowska@umw.edu.pl (A.P.); alicja.kmiecik@umw.edu.pl (A.K.); 2 Department of Ultrastructural Research, Wroclaw Medical University, 50-368 Wroclaw, Poland; marzenna.podhorska-okolow@umw.edu.pl (M.P.-O.); michal.kulus@umw.edu.pl (M.J.K.); 3 Department of Human Biology, Wroclaw University of Health and Sport Sciences, 51-612 Wroclaw, Poland

**Keywords:** periostin (POSTN), non-small-cell lung carcinoma (NSCLC), angiogenesis, pro-angiogenic factors, invasion, cancer-associated fibroblasts (CAFs), tumor microenvironment, cancer, endothelial cells (ECs), extracellular matrix (ECM)

## Abstract

The role of periostin (POSTN) in remodeling the microenvironment surrounding solid tumors and its effect on the tumor cells in non-small-cell lung carcinoma (NSCLC) have not yet been fully understood. The aim of this study was to determine the relationship between POSTN expression (in tumor cells [NSCLC cells] and the tumor stroma) and pro-angiogenic factors (CD31, CD34, CD105, and VEGF-A) and microvascular density (MVD) in NSCLC. In addition, these associations were analyzed in individual histological subtypes of NSCLC (SCC, AC, and LCC) and their correlations with clinicopathological factors and prognosis were examined. Immunohistochemistry using tissue microarrays (TMAs) was used to assess the expression of POSTN (in tumor cells and cancer-associated fibroblasts [CAFs]) and the pro-angiogenic factors. A significant positive correlation was found between the expression of POSTN (in cancer cells/CAFs) and the expression of the analyzed pro-angiogenic factors (CD31, CD34, CD105, and VEGF-A) and MVD in the entire population of patients with NSCLC and individual histological subtypes (AC, SCC). In addition, this study found that POSTN expression (in tumor cells/CAFs) increased with tumor size (pT), histopathological grade (G), and lymph-node involvement (pN). In addition, a high expression of POSTN (in tumor cells and CAFs) was associated with shorter survival among patients with NSCLC. In conclusion, a high expression of POSTN (in cancer cells and CAFs) may be crucial for angiogenesis and NSCLC progression and can constitute an independent prognostic factor for NSCLC.

## 1. Introduction

Cancers constitute a complex and common group of conditions that are one of the leading causes of premature deaths worldwide [1]. The WHO report shows that lung cancer is not only characterized by the highest mortality rate among the 36 types of cancer [2] but also ranks second in terms of worldwide prevalence [2]. These data shed light on the importance of addressing this problem and stress the need for further intensive research into searching for prognostic and predictive markers and new effective methods of prevention and treatment.

Non-small-cell lung carcinoma (NSCLC) is one of the main lung cancer subtypes, accounting for about 85% of all cases of this disease [3,4,5,6]. High mortality rates associated with NSCLC [7] result from many factors, including late diagnosis, rapid disease progression, and the limited effectiveness of traditional treatment methods [6]. Angiogenesis plays a crucial role in the unfavorable prognosis of NSCLC [8]. Defined as the process of new blood-vessel formation [9,10,11], angiogenesis affects the tumor microenvironment, creating favorable conditions for further tumor development and disease progression.

Angiogenesis is a complex process that involves the formation of new capillaries from pre-existing ones [10,11]. In this mechanism, vessels elongate and branch out in response to stimuli generated by pro-angiogenic factors [12]. The main role of angiogenesis is to deliver oxygen and nutrients to cells [9] and to remove waste products [13]. Angiogenesis is essential for physiological processes, such as tissue development, tissue regeneration, and wound healing [14,15]. Neovascularization is also important in the context of diseases, especially oncological conditions [13]. This process contributes to tumor growth and allows cancer cells to spread to other tissues and organs, forming metastases [13]. The formation of new blood vessels is regulated by various factors, one of the main activators being hypoxia [16,17,18]. It begins when angiogenesis-promoting factors (e.g., PIGF, Ang 1, FGF-2, MMP, and Nogo-A) [19,20,21] predominate compared to factors that inhibit it (such as angiostatin, endostatin, or tissue metalloproteinase inhibitors) [22,23,24,25,26].

Many reports draw attention to the role of POSTN as one of the key factors that can significantly affect the process of new blood-vessel formation [27,28], especially in the context of cancer. POSTN is a non-structural extracellular matrix (ECM) protein [29], which is synthesized by both cancer cells [30,31,32] and cancer-associated fibroblasts (CAFs) [27,28,31,32,33]. It plays an essential role in regulating the tissue microenvironment and intercellular communication [1]. The POSTN glycoprotein is involved in many important physiological and pathological biological processes [1]. POSTN participates in the development of the heart [34,35], myocardial remodeling after a coronary event [36,37], the formation of bone and tooth structures [38], and tissue regeneration after injury [39,40]. This protein is mainly present in collagen-rich areas, such as the periosteum, the periodontal ligament, or heart valves [41]. POSTN is thought to reach high levels, especially in cancerous tumors [1,42], ovarian cancer [43], squamous-cell carcinoma of the head and neck [44], or colorectal cancer [45]. POSTN interacts with various receptors, influencing the activation of many intracellular signaling pathways, including TGF-β, PI3K/Akt, Wnt, RhoA/ROCK, NF-κB, MAPK, and JAK [42]. Its increased expression in tumors and its participation in processes such as angiogenesis, migration, invasion, the epithelial–mesenchymal transition (EMT), and the formation of metastases [30,31,46,47,48,49] contribute to the development and progression of many diseases, including cancers. POSTN plays an essential role in the development of new blood vessels by regulating endothelial cell (EC) function in the existing vessels and engaging in the processes of recruitment and differentiation of progenitor cells [50,51,52]. Furthermore, the relationship between the level of POSTN expression and the presence of factors that promote blood-vessel formation (e.g., the VEGF protein family) has already been described in many types of cancer [53,54]. However, in the case of NSCLC, there are fewer analyses [55]. Therefore, conducting new research and considering new concepts should be continued.

The aim of this study was to analyze the correlation of POSTN expression (in cancer cells and CAFs) with pro-angiogenic factors (CD31, CD34, CD105, and VEGF-A) in patients with NSCLC. In addition, we also aimed to determine this relationship for individual histological subtypes of NSCLC (SCC, AC, LCC) and compare the results with the clinical course of the disease in order to isolate prognostic and predictive factors. 

## 2. Materials and Methods

### 2.1. Patient Group

To ensure the statistical power for the detection of less pronounced differences among the study groups, a relatively large cohort was selected for the current study. The study material consisted of 500 cases of NSCLC (248 SCC, 214 AC, and 38 LCC samples from tissue microarrays [TMAs]) and 30 non-malignant lung tissue (NMLT) samples. These were obtained during lung parenchymal resection or lobectomy from patients with NSCLC treated at the Lower Silesian Center for Lung Diseases in Wroclaw between 2007 and 2011. Sampling occurred before the start of treatment, which made it possible to eliminate the influence of therapeutic interventions on the results. The type and histological grade (G) of the tumors were assessed according to the World Health Organization classification criteria [56]. In turn, the pathological stage (TNM) was determined according to the recommendations of the International Association for Lung Cancer Research (IASLC) [57]. All procedures were conducted in accordance with all ethical standards. This study was approved by the Bioethics Committee of Wroclaw Medical University (KB-658/2020). Detailed clinicopathological data of the patients are given in Table 1.

### 2.2. Formation of Tissue Microarrays (TMAs)

Tissue microarrays (TMAs) were constructed from archived lung tissue samples fixed in formalin and embedded in paraffin blocks. Initially, the original paraffin blocks and the slides stained via the H&E method were scanned using the Pannoramic Midi II histological scanner (3D HISTECH, Budapest, Hungary). Next, using the Pannoramic Viewer 1.15.4 software (3D HISTECH, Budapest, Hungary, RRID: SCR_014424), three representative tumor areas were identified for each sample. In the next step, cylindrical fragments (cores) with a diameter of 1.5 mm were extracted from the selected tissue areas using the TMA Grand Master (3D HISTECH, Budapest, Hungary). The cores were placed in a new paraffin block, forming a microarray. This method made it possible to simultaneously perform immunohistochemistry studies on many tissue sections, thus reducing the cost and time needed to perform the procedure [58,59].

### 2.3. Immunohistochemistry 

Immunohistochemical (IHC) reactions were performed using Autostainer Link 48 (Agilent Technologies, Santa Clara, CA, USA). For the IHC reaction, paraffin blocks were cut using a rotational microtome (Leica RM2255, Deer Park, IL, USA) into paraffin sections with a thickness of 4 μm. The following specific primary antibodies were used in the IHC experiments: anti-POSTN rabbit polyclonal antibody (1:200 dilution, NBP1-82472, Novus Biologicals, Littleton, CO, USA), monoclonal mouse anti-VEGF-A antibody (1:50 dilution + linker, VG1 clone, M7273, Agilent Technologies, Santa Clara, CA, USA), monoclonal mouse anti-CD31 antibody (RTU, JC70A clone, IR610, Agilent Technologies, Santa Clara, CA, USA), monoclonal mouse anti-CD34 antibody (RTU, QBEnd/10 clone, IR632, Agilent Technologies, Santa Clara, CA, USA), and rabbit polyclonal anti-CD105 antibody (1:1000 dilution, 10862-1-AP, Proteintech, Manchester, UK). An EnVision FLEX system (Agilent Technologies, Santa Clara, CA, USA) was used to visualize the reactions in accordance with the manufacturer’s instructions.

### 2.4. Assessment of IHC Reactions

To assess the expression levels of POSTN (in tumor cells/tumor stroma) and VEGF-A, the semi-quantitative immunoreactive score (IRS) proposed by Remmele and Stegner was used [60]. This scale takes into account the percentage of stained cells and the intensity of the color reaction. The final score (ranging from 0 to 12) is obtained by multiplying the scores from both categories (Table 2). In our study, an Olympus CX23 light microscope (Olympus, Tokyo, Japan) was used.

The expression of certain pro-angiogenic factors (CD31, CD34, CD105) was also assessed in the NSCLC samples, which required is to visualize the blood vessels. In each cylindrical core of the tissues, the area with the highest blood-vessel density was selected (power field; 400× magnification). The mean number of vessels counted in three histological spots constituted the microvessel density of each case (Weidner method) [61]. The assessment of CD31, CD34, and CD105 expression was performed using the Olympus CX23 light microscope (Olympus, Tokyo, Japan). In addition, the expression of the above factors was also assessed through the Chalkley method [61], which made it possible to eliminate the possibility of counting the same blood vessels twice [62]. The Chalkley analysis was carried out using the Olympus BX-41 light microscope (Olympus, Tokyo, Japan) using a special ocular.

A correlation analysis was also performed between the expression of POSTN (in tumor cells/tumor stroma) and the vascular count. Additionally, we assessed all associations between POSTN expression and clinicopathological features and patient prognosis. TMAs that underwent IHC staining were scanned to create a digital database of histological slides.

### 2.5. Statistical Analyses

Project R version 4.3.2 (The R Foundation for Statistical Computing, Vienna, Austria), Statistica version 13.3 (Tibco Software Inc., Palo Alto, CA, USA), and Jamovi version 2.3 were used for the statistical analyses. Due to the semi-quantitative nature of some variables, non-parametric tests were used. The Mann–Whitney U test was used to compare the data between two groups. The Kruskal–Wallis test with the corresponding post hoc test was used to compared data between three or more groups. Correlation analyses were performed using Spearman’s rank correlation coefficient. Survival was assessed using the Kaplan–Meier method and the assessment of differences between the groups was determined using the log-rank test. The value of POSTN in predicting survival was evaluated using the multivariable linear regression and binomial logistic regression techniques, with stepwise model building being employed to construct the regression models. All statistical analyses were considered significant at *p* < 0.05.

## 3. Results

### 3.1. Expression of POSTN and Pro-Angiogenic Factors (CD31, CD34, CD 105, VEGF-A) in NSCLC Cells and Non-Malignant Lung Tissue (NMLT)

**Figure 1:**
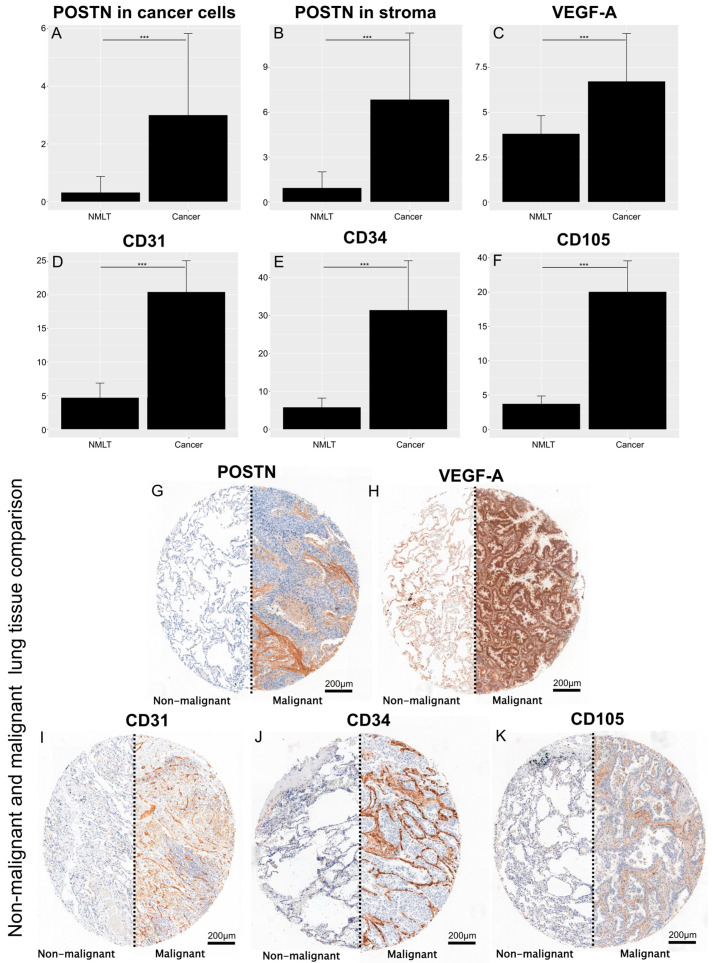
Comparison of the expression of POSTN (in cells (**A**) and in stroma (**B**)) and the expression of pro-angiogenic markers (VEGF-A (**C**); CD31 (**D**); CD34 (**E**); CD105 (**F**)) between non-malignant lung tissue and cancer cells. The significance of the differences was determined using the Mann–Whitney U test. The images in (**G**–**K**) show punches from tissue microarrays illustrating an example of the immunohistochemical (IHC) reaction for each individual protein, as described above. Error bars represent the standard deviation (SD). *** *p* < 0.001.

This study found a significantly higher expression of POSTN in cancer cells and tumor stroma (Figure 1A,B) and a significantly higher expression of pro-angiogenic factors (VEGF-A—Figure 1C; CD31—Figure 1D; CD34—Figure 1E; CD105—Figure 1F) in NSCLC cells compared to NMLT (*** *p* < 0.001; Mann–Whitney U test) (Figure 1). Figure 1G–K show representative tissue microarray sections showing the IHC expression of individual proteins (G-POSTN, H-VEGF-A, I-CD31, J-CD34, and K-CD105) in normal and tumorous lung tissues.

### 3.2. Relationship between Immunohistochemical (IHC) Expression of POSTN in NSCLC and Clinicopathological Data of Patients

**Figure 2:**
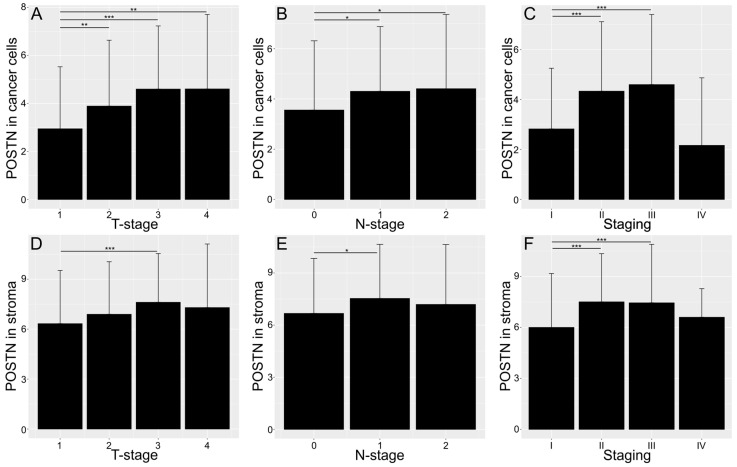
Bar charts showing the expression of POSTN in cells (**A**–**C**) and in stroma (**D**–**F**) across all cancer types with regard to the T-stage (**A**,**D**) and N-stage (**B**,**E**) of the TNM classification as well as the tumor stage (**C**–**F**). The significance of the differences was determined using the Kruskal–Wallis ANOVA test and the differences between individual groups were evaluated using the appropriate post hoc test. Error bars represent the standard deviation (SD). * *p* < 0.05; ** *p* < 0.01; *** *p* < 0.001.

In the NSCLC group, a significantly higher level of POSTN expression was demonstrated in the tumor cells and stroma of pT3 tumors compared to pT1 tumors (*** *p* < 0.001; Figure 2A,D). The analysis also showed that pT2 and pT4 tumors showed a similar pattern of a high POSTN expression in tumor cells in relation to pT1 tumors (** *p* < 0.01; Figure 2A). Additionally, high levels of POSTN expression in tumor cells and tumor stroma correlated with the presence of single metastases to regional lymph nodes (pN1; * *p* < 0.05; Figure 2B,E). In addition, a significant increase in POSTN expression in tumor cells was also associated with many metastases to regional lymph nodes in the overall NSCLC group (pN2; * *p* < 0.05; Figure 2B). This study found that POSTN expression in tumor cells and tumor stroma increased proportionally to the clinical stage of the tumor in the entire study group (*** *p* < 0.001; Figure 2C,F, “Staging”). Statistically significant differences were found in the levels of POSTN expression between clinical stages I and II as well as between I and III (*** *p* < 0.001; Figure 2C,F).

**Figure 3:**
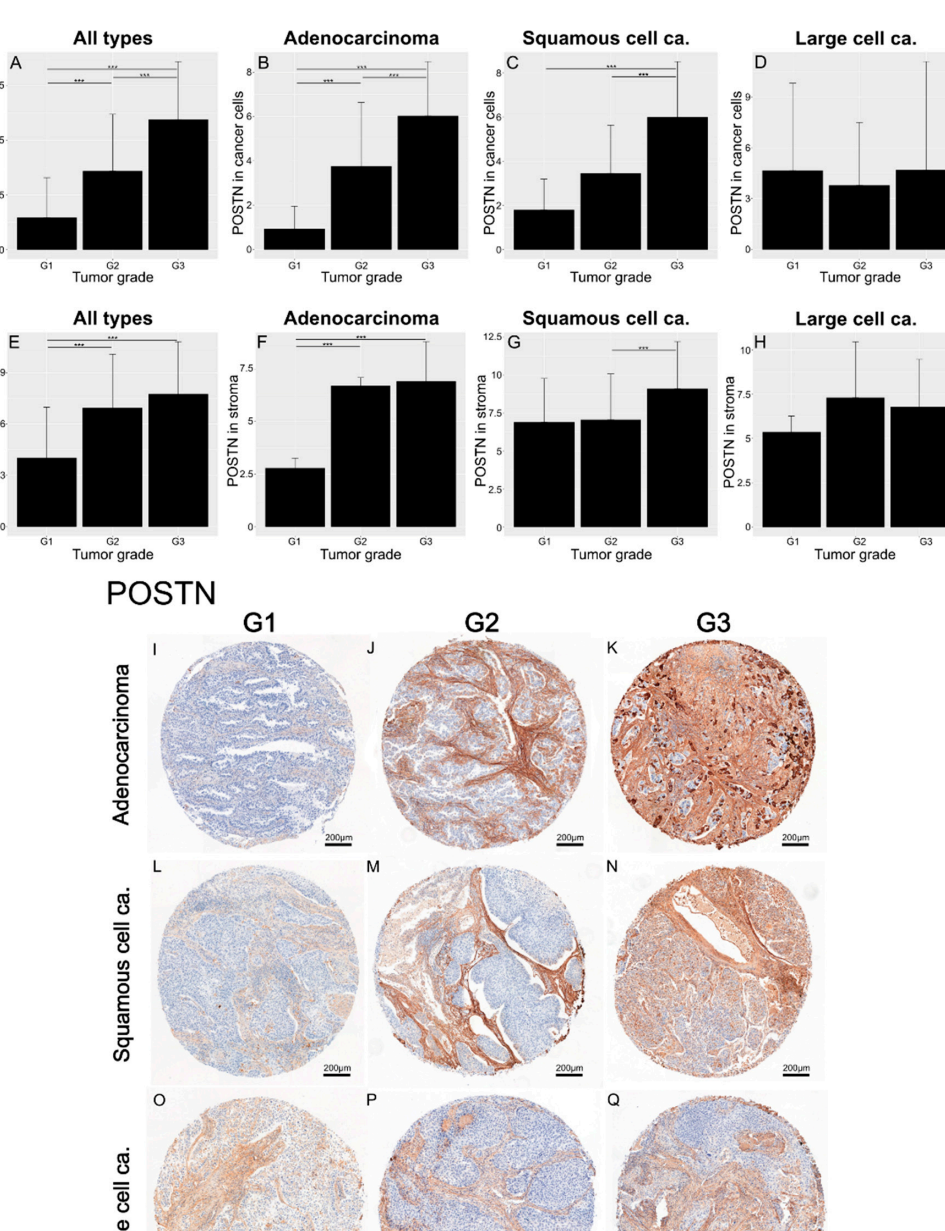
Bar charts showing the expression of POSTN in cells (**A**–**D**) and in stroma (**E**–**H**), across all cancer types (**A**,**E**), in adenocarcinoma (**B**,**F**), and in squamous-cell carcinoma (**C**,**G**), with regard to the tumor grade. The significance of the differences was determined using the Kruskal–Wallis ANOVA test (*p* < 0.001 in bar plots (**A**–**C**,**E**–**G**)), and differences between individual groups were evaluated with the appropriate post hoc test. The results obtained for large-cell carcinoma were statistically insignificant (**D**,**H**). The images in (**I**–**Q**) show representative punches demonstrating the IHC reaction for POSTN in adenocarcinoma (**I**–**K**), squamous-cell carcinoma (**L**–**N**), and large-cell carcinoma (**O**–**Q**) with respect to the tumor grade. Error bars represent the standard deviation (SD). *** *p* < 0.001.

In addition, the results showed an increasing level of POSTN expression in both tumor cells and tumor stroma with an increase in the histological grade (G). Statistically significant differences were noted between G1 tumors and G2 and G3 tumors in the overall NSCLC patient population (*** *p* < 0.001; Figure 3A,E), as well as in the AC subtype (*** *p* < 0.001; Figure 3B,F). Additionally, significant differences were found in the expression of POSTN in cancer cells between G2 and G3 tumors in the entire NSCLC group and in the AC and SCC subtypes (*** *p* < 0.001; Figure 3A–C). In turn, significant differences were found only between G2 and G3 tumors in SCC cases expressing POSTN in the tumor stroma (*** *p* < 0.001; Figure 3G). Figure 3I–Q show tissue microarray sections representing the IHC reactions of POSTN expression in relation to the histological grade in adenocarcinoma (I–K), squamous-cell carcinoma (L–N), and large-cell carcinoma (O–Q). Large-cell carcinoma is a relatively rare subtype of NSCLC. Therefore, the number of LCC cases was most likely insufficient to detect statistically significant differences (Figure 3D,H).

**Figure 4:**
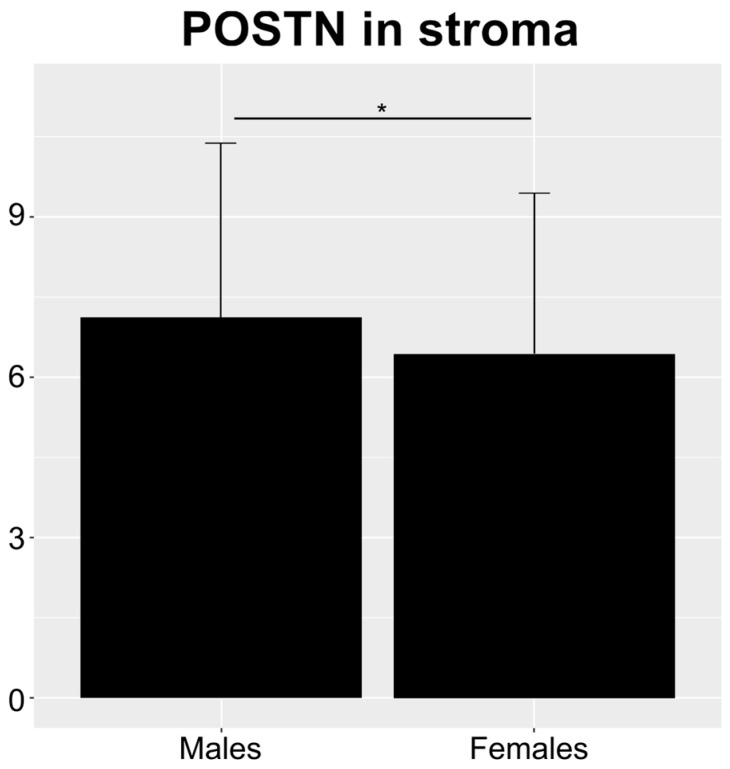
Bar plot showing the only statistically significant difference between males and females (Mann–Whitney U test). Error bars represent the standard deviation (SD). * *p* < 0.05.

Most variables demonstrated no statistically significant differences between males and females (Table 3). However, an exception was observed for POSTN in stroma, which showed differences (Figure 4).

### 3.3. Correlations between POSTN Expression in Cancer Cells and POSTN Expression in NSCLC Stroma

**Figure 5:**
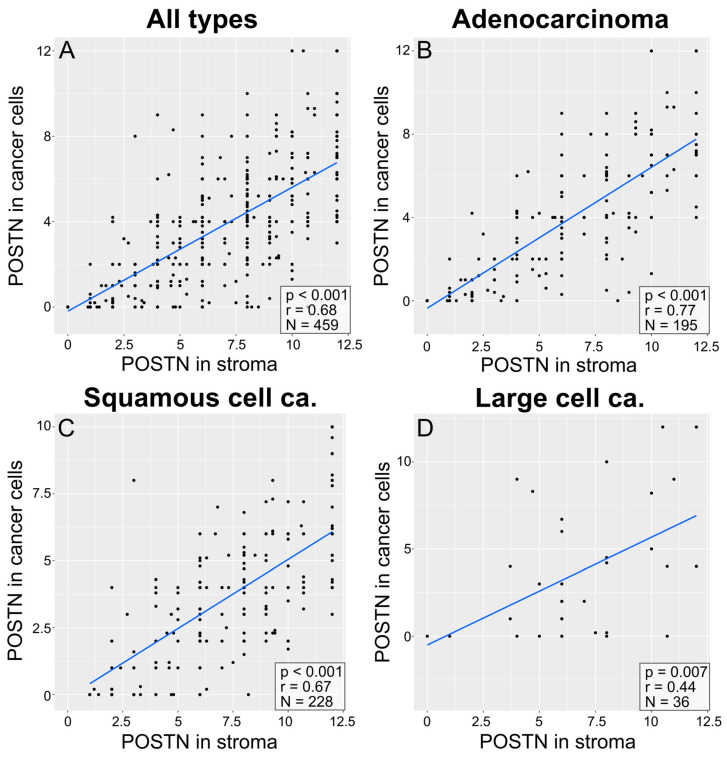
Correlation plots comparing POSTN expression in cells to POSTN expression in stroma, across all cancer types (**A**), in adenocarcinoma (**B**), in squamous-cell carcinoma (**C**), and in large-cell carcinoma (**D**). The plots include *p*-values and R-values (calculated with Spearman’s correlation test) and the number of cases, which can be seen in the lower right corner of each plot.

The correlation analysis showed a significant positive correlation (Spearman’s correlation test) between POSTN expression in tumor cells and its expression in the tumor stroma (r = 0.68; *p* < 0.001; Figure 5A) in the entire group of patients with NSCLC, as well as in individual subtypes: AC (r = 0.77, *p* < 0.001; Figure 5B), SCC (r = 0.67, *p* < 0.001; Figure 5C), and LCC (r = 0.44, *p* = 0.007; Figure 5D).

### 3.4. Correlations of the Expression of Pro-Angiogenic Factors (CD31, CD34, CD105) in NSCLC as Examined with the Methods of Weidner and Chalkley

**Figure 6:**
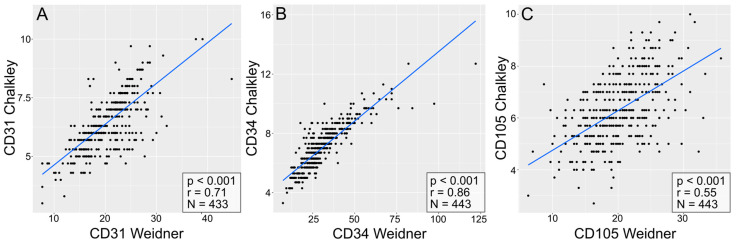
Correlation plots comparing the quantities of CD31-positive (**A**), CD34-positive (**B**), and CD105-positive (**C**) vessels quantified via the Chalkley and Weidner methods. The plots include *p*-values and R-values (calculated with Spearman’s correlation test) and the number of cases, which can be seen in the lower right corner of each plot.

In the entire group of patients with NSCLC, a significant positive correlation (Spearman’s correlation test) was found between the levels of expression observed for different pro-angiogenic factors, i.e., CD31 (r = 0.71, *p* < 0.001; Figure 6A), CD34 (r = 0.86, *p* < 0.001; Figure 6B), and CD105 (r = 0.55, *p* < 0.001; Figure 6C), assessed using the methods of Weidner and Chalkley.

### 3.5. Correlations between POSTN Expression and Pro-Angiogenic Factor Expression in NSCLC

**Figure 7:**
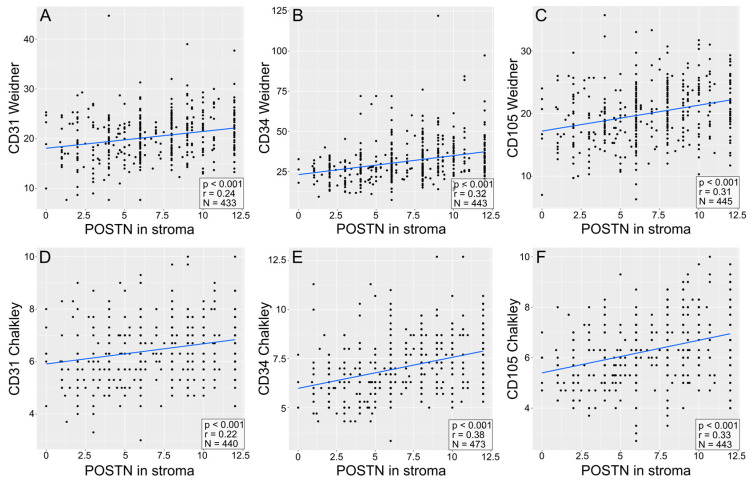
Correlation plots comparing the expression of POSTN in stroma with the expression of pro-angiogenic markers, as quantified via the Weidner (**A**–**C**) or Chalkley (**D**–**F**) method. The pro-angiogenic factors are CD31 (**A**,**D**), CD34 (**B**,**E**), and CD105 (**C**,**F**). The plots include *p*-values and R-values (calculated with Spearman’s correlation test) and the number of cases, which can be seen in the lower right corner of each plot.

**Figure 8:**
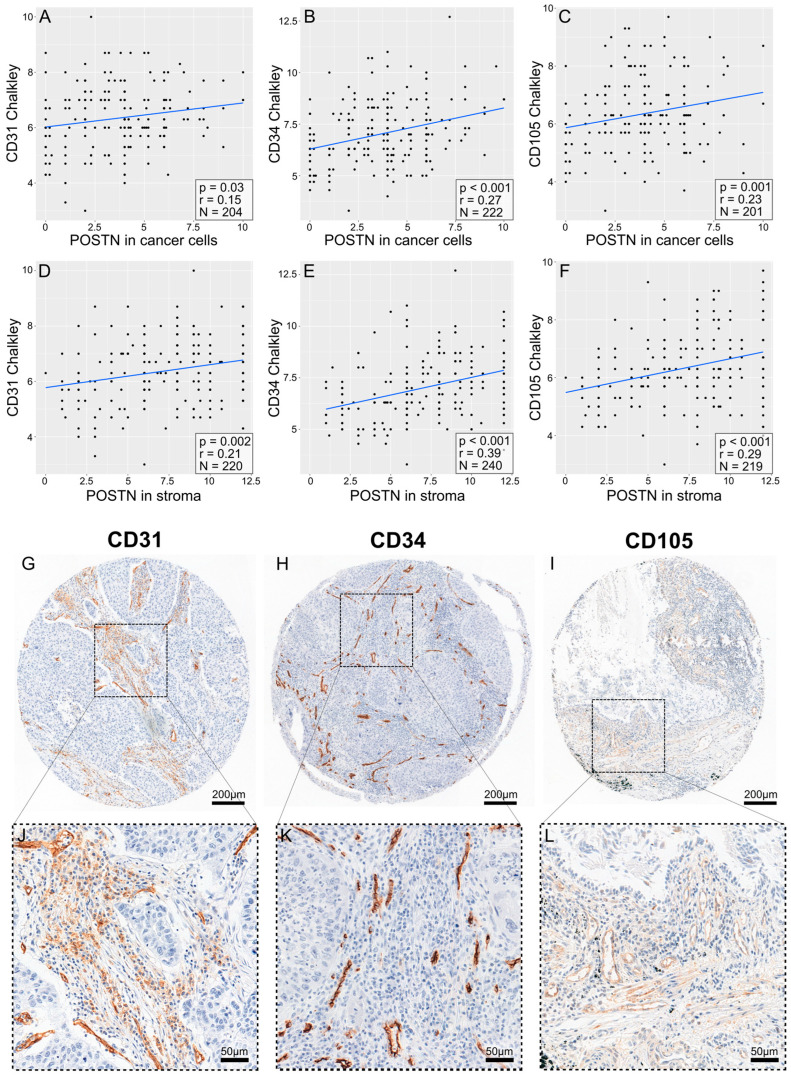
Correlation plots comparing the expression of POSTN in cells (**A**–**C**) and in stroma (**D**–**F**) with the expression of pro-angiogenic markers, as quantified via the Chalkley method, assessed in adenocarcinoma. The pro-angiogenic factors are CD31 (**A**,**D**), CD34 (**B**,**E**), and CD105 (**C**,**F**). The plots include *p*-values and R-values (calculated with Spearman’s correlation test) and the number of cases, which can be seen in the lower right corner of each plot. The images in (**G**–**I**) show punches from tissue microarrays demonstrating an example of the immunohistochemical (IHC) reaction for each individual protein, as previously described. The images in (**J**–**L**) show representative magnified regions of the microarray punches.

**Figure 9:**
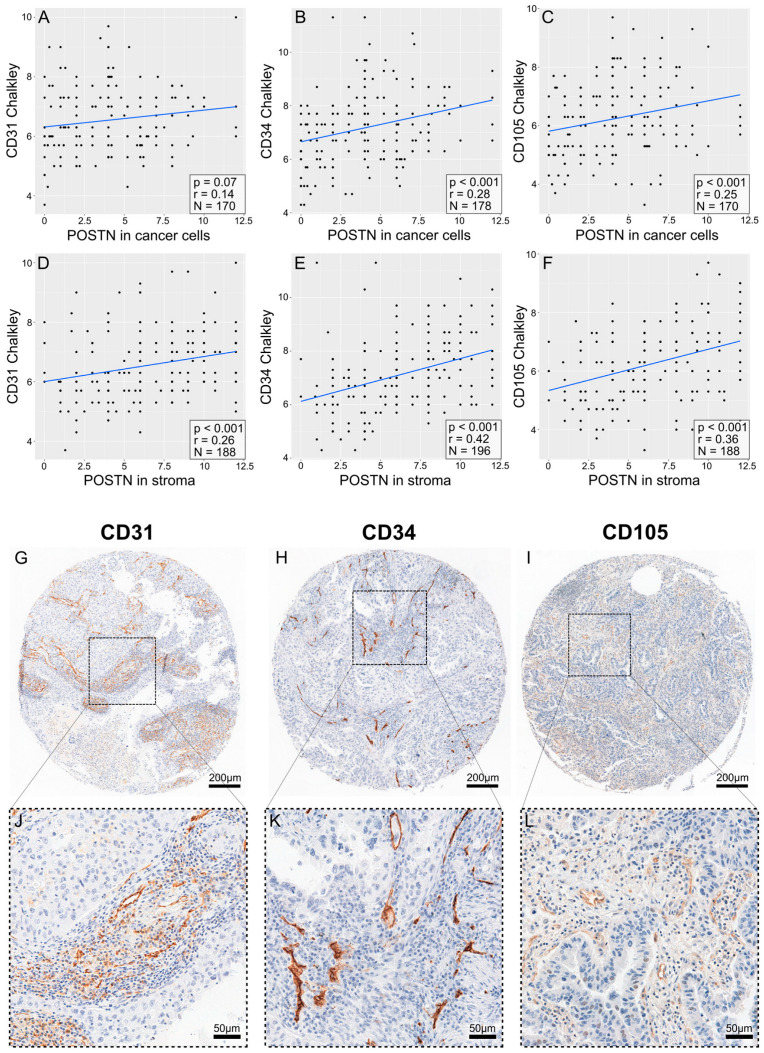
Correlation plots comparing the expression of POSTN in cells (**A**–**C**) and in stroma (**D**–**F**) with the expression of pro-angiogenic markers, as quantified via the Chalkley method, assessed in squamous-cell carcinoma. The pro-angiogenic factors are CD31 (**A**,**D**), CD34 (**B**,**E**), and CD105 (**C**,**F**). The plots include *p*-values and R-values (calculated with Spearman’s correlation test) and the number of cases, which can be seen in the lower right corner of each plot. The images in (**G**–**I**) show punches from tissue microarrays demonstrating an example of the immunohistochemical (IHC) staining for each individual protein, as previously described. The images in (**J**–**L**) show representative magnified regions of the microarray punches.

A significant positive correlation (Spearman’s correlation test) was found between POSTN expression in tumor cells (Table S1) and tumor stroma and the expression of pro-angiogenic factors, i.e., CD31 (Weidner: r = 0.24, *p* < 0.001; Figure 7A) (Chalkley: r = 0.22; *p* < 0.001; Figure 7D), CD34 (Weidner: r = 0.32, *p* < 0.001; Figure 7B) (Chalkley: r = 0.38, *p* < 0.001; Figure 7E), and CD105 (Weidner: r = 0.31, *p* < 0.001; Figure 7C) (Chalkley: r = 0.33, *p* < 0.001; Figure 7F), in the entire group of patients with NSCLC and in the histological subtypes of AC and SCC (Figure 8 and Figure 9, Tables S2 and S3).

**Figure 10:**
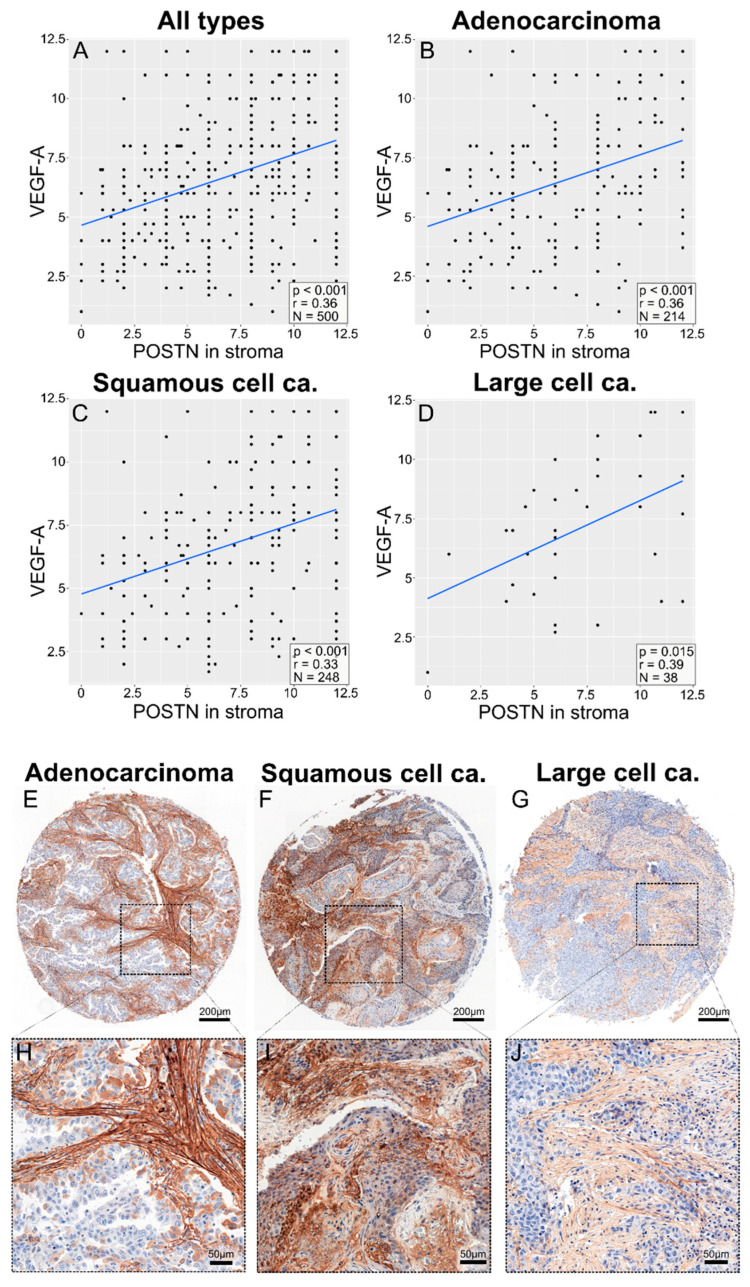
Correlation plots comparing the expression of POSTN in stroma (**A**–**D**) with the expression of VEGF-A, as assessed with the semi-quantitative IRS method, across all cancer types (**A**), in adenocarcinoma (**B**), in squamous-cell carcinoma (**C**), and in large-cell carcinoma (**D**). The images in (**E**–**G**) show punches from tissue microarrays demonstrating an example of the immunohistochemical (IHC) staining for each individual protein, as previously described. The images in (**H**–**J**) show representative magnified regions of the microarray punches.

In addition, a statistically significant positive correlation (Spearman’s correlation test) was found between POSTN expression in cancer cells (Table S4)/in tumor stroma and VEGF-A expression across the entire group of patients with NSCLC (r = 0.36, *p* < 0.001; Figure 10A), as well as in the individual histological subtypes: AC (r = 0.36, *p* < 0.001; Figure 10B), SCC (r = 0.33, *p* < 0.001; Figure 10C), and LCC (r = 0.39, *p* = 0.015; Figure 10D). 

### 3.6. Survival Analysis

**Figure 11:**
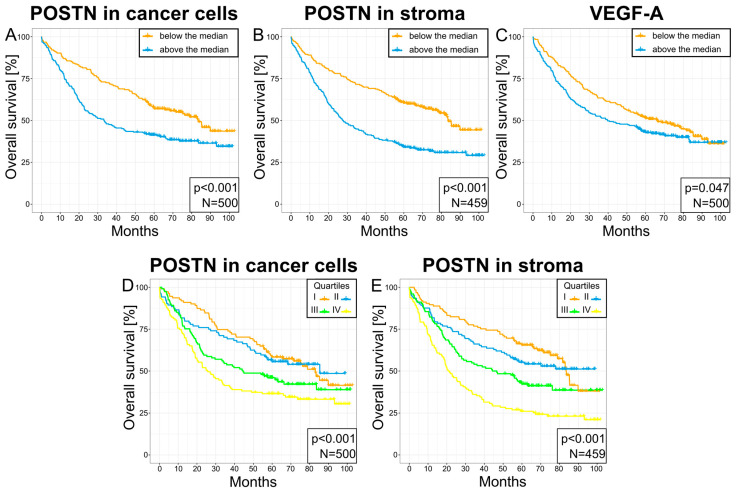
Kaplan–Meier plots of survival for patients expressing POSTN in their cells (**A**), POSTN in their stroma (**B**), and VEGF-A (**C**) at levels below or above the median. The graphs in (**D**,**E**) show the plots of survival for patients with regard to the quartile classifications of the levels of POSTN expression in their cells (**D**) or stroma (**E**). Quartiles are shown in ascending order, with I representing the first 25% of results, II representing 26–50%, III representing 51–75%, and IV representing 76% and above. The significance of the differences was determined with the log-rank test. The *p*-value and the number of cases are given in the lower right corner of each plot.

**Figure 12:**
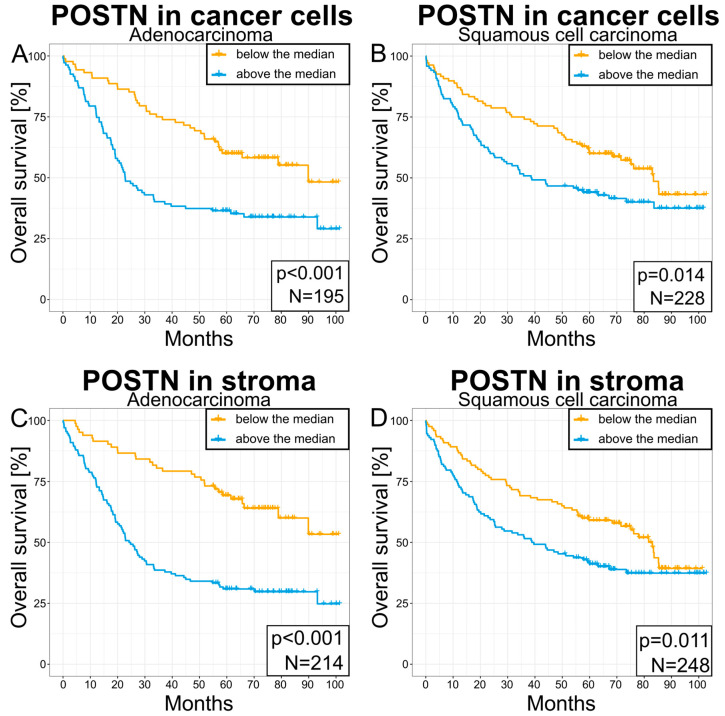
Kaplan–Meier plots of survival for patients with POSTN in their cells (**A**,**B**) or stroma (**C**,**D**) with regard to the tumor type (adenocarcinoma (**A**,**C**) or squamous-cell carcinoma (**B**,**D**)). The significance of the differences was determined using the log-rank test. The *p*-value and the number of cases are given in the lower right corner of each plot.

Survival analysis of these patients with NSCLC showed that those with a high expression of POSTN in their cancer cells and tumor stroma were characterized by significantly shorter survival compared to patients showing a low expression of this glycoprotein. Statistically significant differences in survival were observed in the overall study population of patients with NSCLC (*** *p* < 0.001—tumor cells and tumor stroma, Figure 11A,B) as well as in individual histological subtypes AC (*p* < 0.001—tumor cells and tumor stroma, Figure 12A,C) and SCC (*p* = 0.014—tumor cells, Figure 12B; *p* = 0.011—tumor stroma, Figure 12D). Figure 11D,E show survival curves dependent on POSTN expression localized in the tumor cells (Figure 11D) and tumor stroma (Figure 11E), with the curves divided into quartiles for the entire NSCLC population (*p* < 0.001). In addition, a high expression of VEGF-A was also significantly associated with shorter survival when compared to a low expression of this protein across the entire group of patients with NSCLC (*p* = 0.047; Figure 11C).

### 3.7. Survival Prediction Models

**Figure 13:**
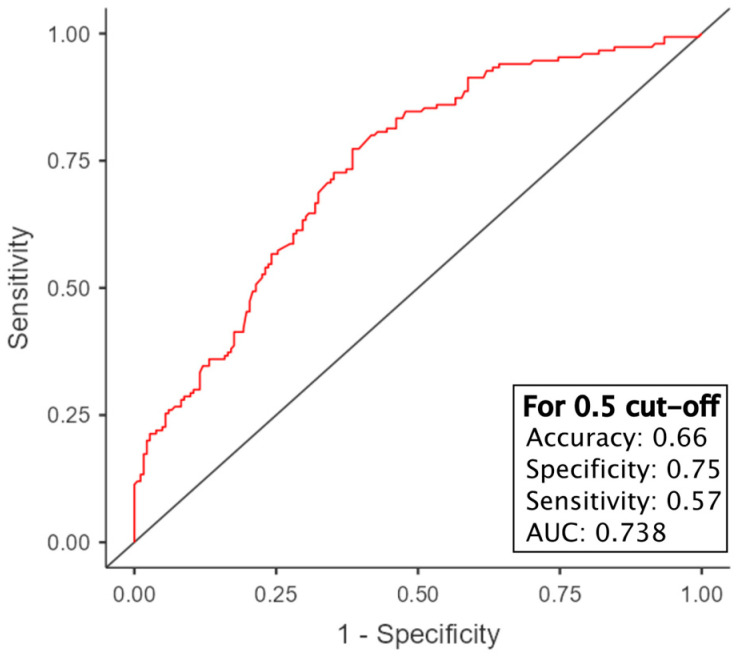
Receiver operating characteristic (ROC) curve demonstrating the sensitivity and specificity of the logistic regression model for predicting patient survival over a five-year period, as detailed in Table 5. This curve illustrates the impact of various cut-off values on the model’s sensitivity and specificity. The area under the curve (AUC) can be regarded as a comprehensive indicator of the model’s overall performance. The values in the lower right quadrant represent the model’s performance when the cut-off is set to 50% of the survival probability.

A further evaluation of the predictive value of POSTN was conducted using multivariable linear and logistic regression models. A linear multivariable regression model was constructed to predict survival days, while a logistic regression model was developed to predict five-year survival. The optimal models are presented in Table 4 and Table 5, respectively. Figure 13 illustrates the receiver operating characteristic (ROC) curve for the logistic regression model, which depicts the effectiveness of this model.

The objective of the above variables shows an R-value of 0.473, indicating that these models can be utilized as reasonably accurate predictors of survival.

The objective of the logistic regression model is to demonstrate the probability of 5-year survival. The current dataset permitted the utilization of POSTN in stroma, the evaluation of CD34 with the Chalkley method, and the assessment of T-stage. The application of these variables to the logistic regression equation with the estimated values given in Table 5 shows the probability of 5-year survival [63]. This is illustrated in Figure 13, which depicts the receiver operating characteristic (ROC) curve. This curve demonstrates the change in specificity and sensitivity in relation to the selected cut-off point, which indicates the likelihood of survival beyond five years.

The model exhibits fair quality, although it is not fully accurate, as evidenced by its area under the curve (AUC) value of 0.738 [64].

To ensure the reliability of the results, observations that were not completed before the five-year period were excluded from this analysis.

## 4. Discussion

Previous studies [65,66] on a larger number of cases of NSCLC assessed the expression of POSTN in tumor cells and CAFs. At the same time, its expression was correlated with clinicopathologic factors. Our results are in line with the findings presented in other papers, which confirms the prognostic significance of POSTN in NSCLC.

As part of our study, we analyzed POSTN expression in tumor cells and tumor stroma and assessed its association with the pro-angiogenic factors (CD31, CD34, CD105, VEGF-A) in NSCLC. As a result, we investigated the relationship between POSTN expression and the formation of new blood vessels, which is directly related to metastasis formation and disease progression.

One of the factors that has an essential impact on the development of metastases is the impaired regulation of new blood-vessel formation [67]. According to Folkman’s hypothesis [68], when a tumor reaches a volume of about 2 mm^3^, it cannot continue growing without developing its vascular system because it cannot obtain enough oxygen and nutrients by diffusion from capillaries [9]. In response to hypoxia, cancer cells stimulate angiogenesis [9,69] via signaling pathways (PI3K-AKT, FAK, Erk/VEGF) [31,70]. It has been shown that POSTN, being a factor stimulating angiogenesis, has the ability to bind to integrin receptors (αvβ3, αvβ5, α6β4) [27,31] located on the surface of cancer cells. This binding induces the activation of the intracellular signaling pathways (PI3K, AKT/PKB, FAK) [30,31], which affects the regulation of processes such as EMT, invasion, angiogenesis, and metastasis formation [30,71,72]. In the FAK pathway, POSTN supports angiogenesis through an increased expression of the VEGFR-2 receptor (located in the ECs), in which its interaction with the αvβ3 integrin receptor plays a crucial role [53]. POSTN also affects the Erk/VEGF pathway, which is activated by binding VEGF-A to its receptor (VEGFR-2), which leads to phosphorylation of phospholipase C gamma (PLC-γ) and triggers the MAPK/ERK pathways [73,74]. Previous studies have demonstrated that increased expression of POSTN in the Erk/VEGF pathway promotes Erk phosphorylation. In addition, a significant positive correlation was found between high POSTN expression in pancreatic cancer and VEGF levels, which suggests an essential role of POSTN in the mechanisms regulating angiogenesis and metastasis development [70]. In addition, it has been proven that a high expression of POSTN in cancer cells promotes their acquisition of resistance to hypoxia [72]. This indicates that POSTN may play an important role in the adaptation of tumors to hypoxic conditions, which is important for further tumor growth and disease development. Our study showed that the number of blood vessels (CD31, CD34, CD105, VEGF-A) in cancerous tissue (NSCLC) was significantly higher compared to healthy lung tissue (NMLT), which is in line with the above description of the mechanisms of neovascularization.

Therefore, the aim of this study was to analyze the correlation of pro-angiogenic factors with POSTN expression (in cancer cells and tumor stroma) in individual histological subtypes of NSCLC. In addition, our study was conducted on a large cohort of patients with NSCLC (N = 500) with consideration given to different histological subtypes and clinicopathological factors. It should be emphasized that research on this subject is scarce and reported in only a few papers [55,75].

Our study found a statistically significant positive correlation between POSTN expression in cancer cells and its expression in the tumor stroma (CAFs) in the entire group of patients and in the histological subtypes analyzed separately (SCC, AC, and LCC). This indicates the possibility of an interplay between cancer cells and stromal cells. Their direct interaction and co-operation with the ECM can lead to further changes in both cell types, which, in turn, can increase tumor invasion in NSCLC, which is stimulated by CAFs [76]. In addition, cancer cells actively use ECM proteins to form a microenvironment that would promote the initiation and progression of the primary tumor and the formation of metastases [77]. Ratajczak-Wielgomas et al. [65] showed that during carcinogenesis, POSTN promoted the migration of cancer cells by regulating the interactions between them and the tumor microenvironment, which may be important for cancer progression.

Studies on cancer neovascularization often use immunohistochemical EC markers (CD31, CD34, CD105) to identify newly formed microvessels in diseased tissues (in this case, NSCLC) [78,79,80,81,82,83,84]. There is no single universal marker for application in all cases. Recent studies comparing these factors in the context of different types of cancer [78,79,80,81] suggest that the use of CD105 as a marker of microvascular density (MVD) may provide better prognostic indicators of disease progression and prognosis than CD31 or CD34 in many types of cancer [78,79,82,83,84], including NSCLC. According to Tanaka et al. [84], CD34, which is commonly used to assess angiogenesis, can stain newly formed vessels and also visualize normal vessels in the tumor tissue, which can make the results difficult to interpret. In turn, CD105 specifically stains ECs involved in the formation of new vessels. Therefore, it has been suggested that the CD105 antibody may be better for assessing angiogenesis, especially in the case of NSCLC. VEGF-A is considered one of the most important pro-angiogenic factors [85,86] among the proteins of its family. It is synthesized by many different types of normal cells, including ECs, vascular smooth-muscle cells, monocytes/macrophages, fibroblasts, cancer cells, and CAFs [87]. By stimulating ECs to increase nitric oxide secretion [88], this factor increases the permeability of blood vessels and remodels their structure, thus activating proteolytic enzymes [1]. This is particularly crucial in neovascularization, which is essential in cancer development and progression [1], including NSCLC [55].

Our results show a statistically significant positive correlation between POSTN expression in tumor cells and tumor stroma and the expression of the analyzed pro-angiogenic factors (CD31, CD34, CD105, and VEGF-A) and MVD in the entire population of patients with NSCLC and in individual histological subtypes (AC/SCC). This stresses the relationship between POSTN expression and angiogenesis, invasion, and metastasis formation.

Our study has also demonstrated a relationship between POSTN expression and some clinicopathological data in patients with NSCLC. POSTN expression increased proportionally with tumor size (pT), histological grade (G), and lymph node involvement (pN). In addition, a statistically significant relationship was found between high POSTN expression and shorter survival in patients with NSCLC. Our results clearly indicate that the level of POSTN expression increases with higher clinical stages of NSCLC. These conclusions are consistent with the results of Wu et al. [75] and Takanami et al. [55]. It should be emphasized, however, that our study is based on the analysis of a significantly larger cohort of patients (N = 500), as well as a broader spectrum of the analyzed pro-angiogenic factors (CD31, CD34, CD105, and VEGF-A), as opposed to the relatively small number of patients with NSCLC (N = 88) and the single marker (von Willebrand factor antigen; F8RA) assessed in the study conducted by Takanami et al. [55]. Our findings are consistent with the observations of Wu et al. [75], who found that a high expression of POSTN in cancer cells (NSCLC cells) directly affected tumor progression, including the growth of new blood vessels and the development of metastases. Additionally, Soltermann et al. [89] confirmed that POSTN expression in NSCLC was significantly associated with a more advanced disease and larger tumor size, which stressed the importance of POSTN as a factor influencing cancer dynamics. A similar topic was also analyzed by Zhu et al. [90], who investigated MVD in ovarian tumor xenografts in mice and observed CD31 expression. They reported that in the analyzed material with a high POSTN expression in cancer cells, higher vascular density was found, which suggests the role of the POSTN glycoprotein in angiogenesis. Similar conclusions in the case of hepatocellular carcinoma were reached by Jang et al. [91]. These authors also proved that high levels of POSTN expression were associated with increased microvascular infiltration, advanced disease stages, and poor prognoses. Similar conclusions were proposed by Lv et al. [92], who showed that a high expression of POSTN in hepatocellular carcinoma was correlated with a higher VEGF expression and a higher MVD compared to tumors negatively expressing this protein [92]. A convergent hypothesis in the case of squamous-cell esophageal cancer was proposed by Wang et al. [50]. They demonstrated that cancerous tissues with high levels of POSTN expression were characterized by a higher level of VEGF expression and a higher MVD compared to tumors with a low expression of this glycoprotein. Furthermore, a high expression of POSTN was strongly associated with lymph-node metastasis, tumor differentiation, venous infiltration, tumor progression, and angiogenesis [50]. Similar conclusions were provided by Morra et al. [93], who showed that a higher expression of POSTN in renal cell carcinoma was correlated with an advanced cancer stage, the presence of lymph-node metastases, and a worse prognosis. In addition, one in vivo study [70] showed that decreasing POSTN levels in pancreatic cancer cells inhibited tumor growth, reduced VEGF expression, and reduced angiogenesis and metastasis in mouse models. Similarly, Zhou et al. [94] showed that the inhibition of POSTN expression in glioma stem cells inhibited tumor growth and prolonged murine survival.

In addition, our study demonstrated a statistically significant relationship between high levels of POSTN expression in tumor cells and tumor stroma and shorter survival in patients with NSCLC overall and in individual histological subtypes of NSCLC, namely AC and SCC. We showed that an increase in the expression of the pro-angiogenic factor VEGF-A was associated with significantly shorter survival in the entire population of patients with NSCLC. Takanami et al. [55] showed a significant positive correlation between POSTN expression in cancer cells and shorter survival in patients with NSCLC with a low expression of this protein, which is in line with our findings. It should be noted, however, that our study was conducted on a significantly larger cohort of patients with NSCLC compared to the study of Takanami et al. [55]. In turn, Hong et al. [95] showed that high levels of POSTN expression in the tumor stroma were significantly associated with shorter overall survival in patients with NSCLC. Additionally, Soltermann et al. [89] found that a high expression of POSTN in the tumor stroma could be an independent prognostic factor in patients with NSCLC. Similar conclusions were also presented by Xu et al. [96], who showed that serum POSTN concentrations in patients with NSCLC (tested via ELISAs) were significantly elevated compared to those in patients with a mild lung disease and healthy controls. Further analysis showed that high serum POSTN levels were significantly associated with shorter progression-free survival and overall survival in patients with NSCLC. They suggested that serum POSTN levels could become a new diagnostic and prognostic marker for patients with NSCLC. Similarly, Zhang et al. [97] showed that serum POSTN levels had the potential to predict the efficacy of chemotherapy in patients with NSCLC [97] and could be used as an independent prognostic factor in this type of cancer. Similarly, in their in vitro study, Wu et al. [75] observed that a high expression of POSTN in cancer cells (A549) was significantly correlated with increased proliferation, migration, and invasion and reduced sensitivity of the cancer cells to chemotherapeutics. Wu et al. [75] highlighted that the POSTN glycoprotein could be an independent prognostic factor and even a potential therapeutic target [75] in NSCLC, which is in line with our findings.

In conclusion, our study has shown that POSTN could play a significant role in NSCLC, promoting angiogenesis and influencing tumor progression. The activation of crucial signaling pathways (PI3K/AKT and FAK) through the interaction of POSTN with integrin receptors in cancer cells directly contributes to increased invasion, proliferation, and cell resistance to apoptosis and hypoxia, as well as to the development and maintenance of the microenvironment surrounding the tumor, thus promoting the formation of metastases. In addition, the impact of POSTN on angiogenesis, especially through its regulation of the Erk/VEGF pathway, is a crucial element enabling the tumor to continue its growth and metastasis. Therefore, these observations confirm the important role of POSTN in the mechanism of angiogenesis and stress its potential as a possible prognostic biomarker in NSCLC.

## 5. Conclusions

The data analysis conducted as part of our study showed a significant positive correlation between the expression of POSTN in tumor cells and tumor stroma (CAFs) and the levels of their expression of pro-angiogenic factors (CD31, CD34, CD105, and VEGF-A) and increased microvascular density in NSCLC compared to NMLT. By interacting with the growth factor VEGF-A and binding to EC integrins (αvβ3, αvβ5, and α6β4) and cancer cells, POSTN activates intracellular signaling pathways (PI3K, AKT/PKB, FAK, and Erk/VEGF), which leads to increased cell survival, angiogenesis, invasion, and metastasis. In addition, a significant relationship was found between POSTN expression and the clinicopathologic data in patients with NSCLC. A high expression of POSTN in tumor cells and tumor stroma may act as an independent prognostic factor in patients with NSCLC and in relation to its histological subtypes (namely SCC and AC). Further research on POSTN related to the development of new targeted therapies for NSCLC is warranted.

## Figures and Tables

**Table 1:** Clinicopathological data of patients with NSCLC.

Variables	NSCLC N = 500 (100%)	SCC N = 248 (49.6%)	AC N = 214 (42.8%)	LCC N = 38 (7.6%)
Age (median 62): <62, ≥62	<62—N = 224 (44.8%) ≥62—N = 276 (55.2%)	<62—N = 97 (39.11%) ≥62—N = 151 (60.89%)	<62—N = 106 (49.53%) ≥62—N = 108 (50.47%)	<62—N = 21 (55.26%) ≥62—N = 17 (44.74%)
Sex: males/females	males—N = 365 (73%) females—N = 135 (27%)	males—N = 196 (79.03%) females—N = 52 (20.97%)	males—N = 139 (64.95%) females—N = 75 (35.05%)	males—N = 30 (78.95%) females—N = 8 (21.05%)
Size of the primary tumor (pT): pT1, pT2, pT3, pT4	pT1—N = 131 (26.2%) pT2—N = 223 (44.6%) pT3—N = 97 (19.4%) pT4—N = 49 (9.8%)	pT1—N = 67 (27.01%) pT2—N = 101 (40.73%) pT3—N = 54 (21.78%) pT4—N = 26 (10.48%)	pT1—N = 53 (24.76%) pT2—N = 105 (49.07%) pT3—N = 38 (17.76%) pT4—N = 18 (8.41%)	pT1—N = 11 (28.94%) pT2—N = 17 (44.74%) pT3—N = 5 (13.16%) pT4—N = 5 (13.16%)
Involvement of regional lymph nodes in NSCLC cells (pN): pN0, pN1, pN2	pN0—N = 321 (64.2%) pN1—N = 96 (19.2%) pN2—N = 83 (16.6%)	pN0—N = 160 (64.52%) pN1—N = 62 (25%) pN2—N = 26 (10.48%)	pN0—N = 135 (63.08%) pN1—N = 29 (13.56%) pN2—N = 50 (23.36%)	pN0—N = 26 (68.42%) pN1—N = 5 (13.16%) pN2—N = 7 (18.42%)
Presence (or absence) of distant metastases (M): pM0, pM1	pM0—N = 497 (99.4%) pM1—N = 3 (0.6%)	pM0—N = 248 (100%)	pM0—N = 211 (98.60%) pM1—N = 3 (1.40%)	pM0—N = 38 (100%)
Histological grade (G): G1, G2, G3	G1—N = 24 (4.8%) G2—N = 383 (76.6%) G3—N = 93 (18.6%)	G1—N = 6 (2.42%) G2—N = 206 (83.06%) G3—N = 36 (14.52%)	G1—N = 16 (7.48%) G2—N = 145 (67.76%) G3—N = 53 (24.76%)	G1—N = 2 (5.26%) G2—N = 32 (84.21%) G3—N = 4 (10.53%)
Cancer stage: I, II, III, IV	I—N = 186 (37.2%) II—N = 164 (32.8%) III—N = 147 (29.4%) IV—N = 3 (0.6%)	I—N = 93 (37.5%) II—N = 90 (36.29%) III—N = 65 (26.21%)	I—N = 79 (36.92%) II—N = 62 (28.97%) III—N = 70 (32.71%) IV—N = 3 (1.4%)	I—N = 14 (36.84%) II—N = 12 (31.58%) III—N = 12 (31.58%)

**Table 2:** IRS scale.

A—The Percentage of Stained Cells	B—The Intensity of the Color Reaction
0 pts—no stained cells	0 pts—no reaction
1 pt < 10% of stained cells	1 pt—weak reaction
2 pts—10–50% of stained cells	2 pts—moderate reaction
3 pts—51–80% of stained cells	3 pts—strong reaction
4 pts > 80% of stained cells	
IRS (A × B): 0–12	

IRS rankings: 0–1—negative, 2–3—poor expression, 4–8—moderate expression, 9–12—strong expression.

**Table 3:** Mann–Whitney U test results for the differences between males and females. Only POSTN in stroma yielded statistically significant results.

Variable	*p*-Value
CD 105 Chalkley	0.840
CD 105 Weidner	0.656
CD 34 Chalkley	0.817
CD 34 Weidner	0.976
CD 31 Chalkley	0.816
CD 31 Weidner	0.195
VEGF-A	0.611
POSTN in cells	0.663
POSTN in stroma	0.032

**Table 4:** Linear regression model constructed for the purpose of predicting survival days. It was determined that other predictors were not valid. The model yielded an R-value of 0.473 when the variables presented below were used.

Predictor	Estimate	SE	t	*p*
Intercept	2851.3	249.8	11.42	<0.001
POSTN in stroma	−77.7	15.0	−5.19	<0.001
CD 34 Chalkley	−70.9	34.7	−2.04	0.042
T-stage:				
2–1	−224.8	110.3	−2.04	0.042
3–1	−545.2	134.5	−4.05	<0.001
4–1	−593.8	159.1	−3.73	<0.001
N-stage:				
1–0	−189.3	114.8	−1.65	0.100
2–0	−521.3	119.2	−4.37	<0.001

**Table 5:** Logistic regression model constructed for the prediction of 5-year survival. It was determined that other predictors were not significant. Measures of model effectiveness are shown on the ROC curve (Figure 13).

Predictor	Estimate	SE	Z	*p*
Intercept	3.402	0.8020	4.24	<0.001
POSTN in stroma	−0.21	0.0404	−5.20	<0.001
CD 31 Chalkley	−0.227	0.1128	−2.01	0.044
T-stage:				
2–1	−0.558	0.3019	−1.85	0.065
3–1	−1.201	0.3670	−3.27	0.001
4–1	−1.635	0.4897	−3.34	<0.001

## Data Availability

The raw data and the analysis methods used in this study will be made available to other researchers upon reasonable request for the purpose of reproducing the results reported here in their laboratories. To access the protocols or datasets, contact katarzyna.ratajczak-wielgomas@umw.edu.pl.

## Supplementary Materials

The following supporting information can be downloaded at https://www.mdpi.com/article/10.3390/cells13171406/s1: Table S1: The correlation between POSTN expression in cancer cells and pro-angiogenic factors (CD31, CD34, CD105) in the entire NSCLC patient cohort as analyzed using the Spearman method. Statistically significant results (*p* < 0.05) are indicated in red. Table S2: The Spearman correlation between POSTN expression (in both cancer cells and tumor stroma) and pro-angiogenic factors (CD31, CD34, CD105) in adenocarcinoma as analyzed using the Weidner method. Statistically significant results (*p* < 0.05) are indicated in red. Table S3: The Spearman correlation between POSTN expression (in both cancer cells and tumor stroma) and pro-angiogenic factors (CD31, CD34, CD105) in squamous-cell carcinoma as analyzed using the Weidner method. Statistically significant results (*p* < 0.05) are indicated in red. Table S4: The Spearman correlation between POSTN expression in cancer cells and the pro-angiogenic factor VEGF-A as analyzed for all cancer types and specific histological subtypes. The semi-quantitative IRS scale was employed to analyze all cancer types, including AC (adenocarcinoma), SCC (squamous-cell carcinoma), and LCC (large-cell carcinoma) cases. Statistically significant results (*p* < 0.05) are indicated in red.
