# Correction: Wasik et al. Correlation Between Periostin Expression and Pro-Angiogenic Factors in Non-Small-Cell Lung Carcinoma. *Cells* 2024, *13*, 1406

**DOI:** 10.3390/cells14191527

**Published:** 2025-09-30

**Authors:** Adrian Wasik, Marzenna Podhorska-Okolow, Piotr Dziegiel, Aleksandra Piotrowska, Michal Jerzy Kulus, Alicja Kmiecik, Katarzyna Ratajczak-Wielgomas

**Affiliations:** 1 Division of Histology and Embryology, Department of Human Morphology and Embryology, Wroclaw Medical University, 50-368 Wroclaw, Poland; adrian.wasik@student.umw.edu.pl (A.W.); piotr.dziegiel@umw.edu.pl (P.D.); aleksandra.piotrowska@umw.edu.pl (A.P.); alicja.kmiecik@umw.edu.pl (A.K.); 2 Department of Ultrastructural Research, Wroclaw Medical University, 50-368 Wroclaw, Poland; marzenna.podhorska-okolow@umw.edu.pl (M.P.-O.); michal.kulus@umw.edu.pl (M.J.K.); 3 Department of Human Biology, Wroclaw University of Health and Sport Sciences, 51-612 Wroclaw, Poland

In the original publication [1], there was a mistake in Figures 3 and 10 as published. We attach an updated Figure 3 (E, G, H, J, O, and P,) including an image which better presents G2 adenocarcinoma. The corrected Figure 3 appears below. POSTN is a glycoprotein that presents expression in both cancer cells and tumor stroma cells. 

Therefore we attach an updated Figure 10 (G and J) with the correct images, showing expression only in stroma cells. The corrected Figure 10 appears below. 

**Figure 10:**
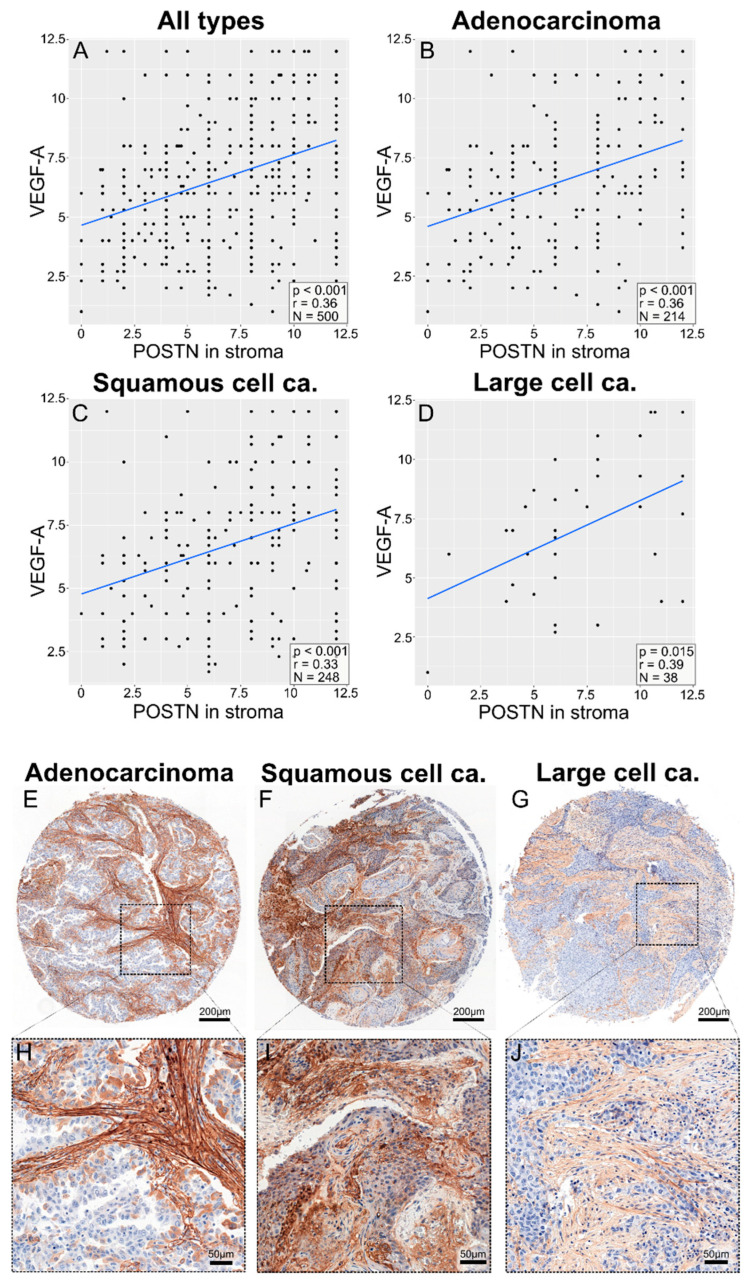
Correlation plots comparing the expression of POSTN in stroma (**A**–**D**) with the expression of VEGF-A, as assessed with the semi-quantitative IRS method, across all cancer types (**A**), in adenocarcinoma (**B**), in squamous-cell carcinoma (**C**), and in large-cell carcinoma (**D**). The images in (**E**–**G**) show punches from tissue microarrays demonstrating an example of the immunohistochemical (IHC) staining for each individual protein, as previously described. The images in (**H**–**J**) show representative magnified regions of the microarray punches.

The authors state that the scientific conclusions are unaffected. This correction was approved by the Academic Editor. The original publication has also been updated.

## Figures and Tables

**Figure 3:**
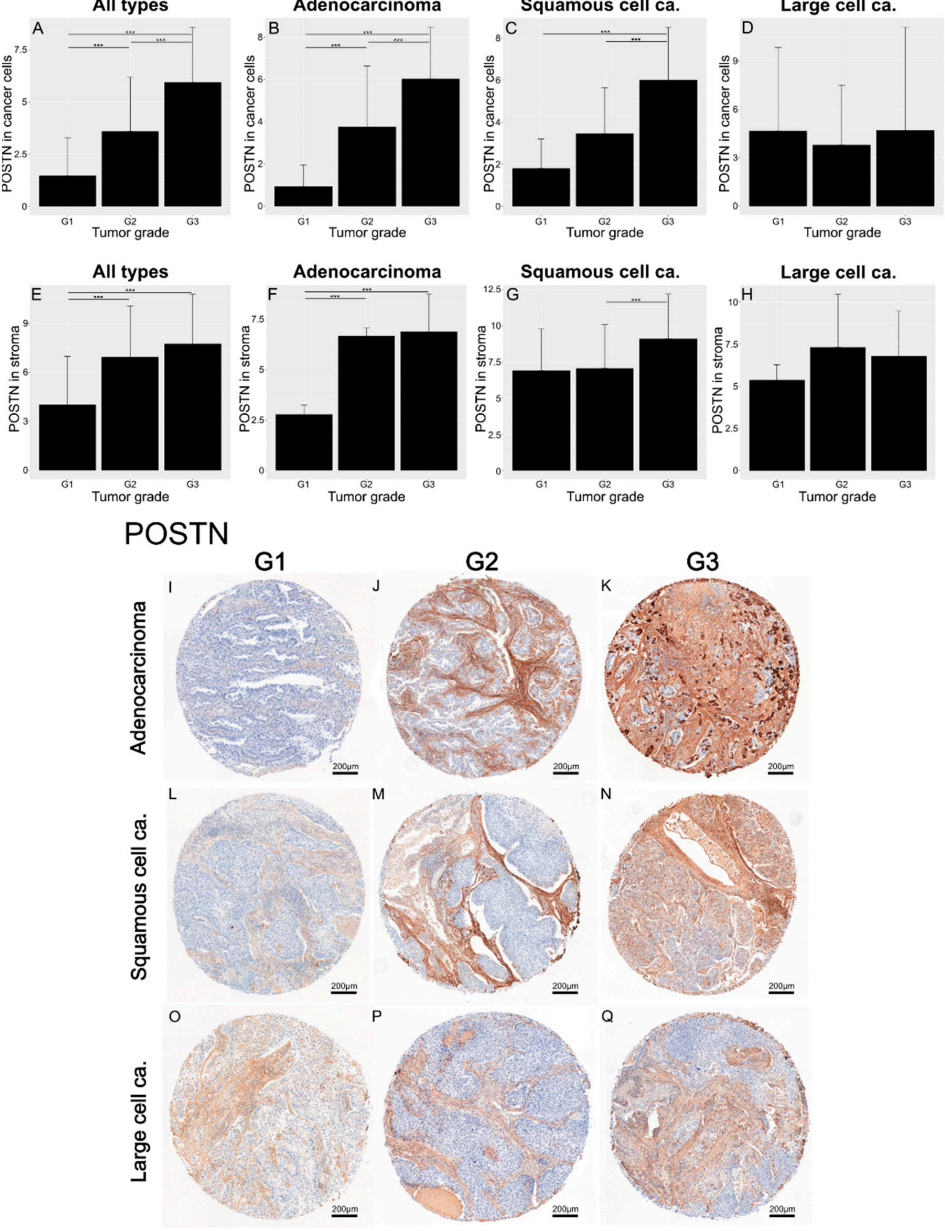
Bar charts showing the expression of POSTN in cells (**A**–**D**) and in stroma (**E**–**H**), across all cancer types (**A**,**E**), in adenocarcinoma (**B**,**F**), and in squamous-cell carcinoma (**C**,**G**), with regard to the tumor grade. The significance of the differences was determined using the Kruskal–Wallis ANOVA test (*p* < 0.001 in bar plots (**A**–**C**,**E**–**G**)), and differences between individual groups were evaluated with the appropriate post hoc test. The results obtained for large-cell carcinoma were statistically insignificant (**D**,**H**). The images in (**I**–**Q**) show representative punches demonstrating the IHC reaction for POSTN in adenocarcinoma (**I**–**K**), squamous-cell carcinoma (**L**–**N**), and large-cell carcinoma (**O**–**Q**) with respect to the tumor grade. Error bars represent the standard deviation (SD). *** *p* < 0.001.
