# Supplementary material for: Correlation Between Periostin Expression and Pro-Angiogenic Factors in Non-Small-Cell Lung Carcinoma
Source: Cells. 2024 Aug 23;13(17):1406. doi: 10.3390/cells13171406 (PMC11394527; doi:10.3390/cells13171406)
Supplement: Supplementary file 1 [file cells-13-01406-s001.zip › cells-3126439-supplementary.pdf]

Table S1. The correlation between POSTN expression in cancer cells and pro-angiogenic factors (CD31, CD34, CD105) in the entire NSCLC patient cohort was analysed using the Spearman method. Statistically significant results ($p < 0.05$) are indicated in red.

Pair of Variables	**All Cancers**		
	Valid N	Spearman R	p-value
POSTN in cells & CD 31 Weidner	398	0.16	0.0016
POSTN in cells & CD 31 Chalkley	404	0.12	0.0164
POSTN in cells & CD 34 Weidner	409	0.21	<0.001
POSTN in cells & CD 34 Chalkley	435	0.25	<0.001
POSTN in cells & CD 105 Weidner	407	0.21	<0.001
POSTN in cells & CD 105 Chalkley	405	0.21	<0.001

Table S2. The Spearman correlation between POSTN expression in both cancer cells and tumor stroma with pro-angiogenic factors (CD31, CD34, CD105) in adenocarcinomas was also analyzed using the Weidner method. Statistically significant results ($p < 0.05$) are indicated in red.

Pair of Variables	**Adenocarcinoma**		
	Valid N	Spearman R	p-value
POSTN in stroma & CD 31 Weidner	188	0.24	<0.001
POSTN in stroma & CD 34 Weidner	186	0.33	<0.001
POSTN in stroma & CD 105 Weidner	189	0.35	<0.001
POSTN in cells & CD 31 Weidner	170	0.16	0.032
POSTN in cells & CD 34 Weidner	170	0.27	<0.001
POSTN in cells & CD 105 Weidner	171	0.32	<0.001

Table S3. The Spearman correlation between POSTN expression in both cancer cells and tumor stroma with pro-angiogenic factors (CD31, CD34, CD105) in squamous cells carcinoma was also analyzed using the Weidner method. Statistically significant results ($p < 0.05$) are indicated in red.

Pair of Variables	**squamous cell carcinoma**		
	Valid N	Spearman R	p-value
POSTN in stroma & CD 31 Weidner	214	0.25	<0.001
POSTN in stroma & CD 34 Weidner	224	0.34	<0.001
POSTN in stroma & CD 105 Weidner	219	0.26	<0.001
POSTN in cells & CD 31 Weidner	199	0.17	0.019
POSTN in cells & CD 34 Weidner	208	0.20	0.0035
POSTN in cells & CD 105 Weidner	201	0.14	0.043

Table S4. The Spearman correlation between POSTN expression in cancer cells and the pro-angiogenic factor VEGF-A was analyzed for all cancer types and in specific histological subtypes. The semi-quantitative IRS scale was employed to analyze all cancer types, including AC (adenocarcinoma), SCC (squamous cell carcinoma) and LCC (large cell carcinoma) cases. Statistically significant results ($p < 0.05$) are indicated in red.

Pair of Variables	**All Cancers**		
	Valid N	Spearman R	p-value
POSTN in cells & VEGF-A IRS	459	0.25	<0.001

Pair of Variables	**Adenocarcinoma**		
	Valid N	Spearman R	p-value
POSTN in cells & VEGF-A IRS	195	0.26	<0.001

Pair of Variables	**Squamous cell carcinoma**		
	Valid N	Spearman R	p-value
POSTN in cells & VEGF-A IRS	228	0.3	<0.001

Pair of Variables	**Large cell carcinoma**		
	Valid N	Spearman R	p-value
POSTN in cells & VEGF-A IRS	36	0.06	0.71
